# Comprehensive benchmarking and ensemble approaches for metagenomic classifiers

**DOI:** 10.1186/s13059-017-1299-7

**Published:** 2017-09-21

**Authors:** Alexa B. R. McIntyre, Rachid Ounit, Ebrahim Afshinnekoo, Robert J. Prill, Elizabeth Hénaff, Noah Alexander, Samuel S. Minot, David Danko, Jonathan Foox, Sofia Ahsanuddin, Scott Tighe, Nur A. Hasan, Poorani Subramanian, Kelly Moffat, Shawn Levy, Stefano Lonardi, Nick Greenfield, Rita R. Colwell, Gail L. Rosen, Christopher E. Mason

**Affiliations:** 1 Tri-Institutional Program in Computational Biology and Medicine, New York, NY USA; 2 Department of Physiology and Biophysics, Weill Cornell Medicine, New York, NY 10021 USA; 3 The HRH Prince Alwaleed Bin Talal Bin Abdulaziz Alsaud Institute for Computational Biomedicine, New York, NY 10021 USA; 4 Department of Computer Science and Engineering, University of California, Riverside, CA 92521 USA; 5 School of Medicine, New York Medical College, Valhalla, NY 10595 USA; 6 Accelerated Discovery Lab, IBM Almaden Research Center, San Jose, CA 95120 USA; 7 One Codex, Reference Genomics, San Francisco, CA 94103 USA; 8 University of Vermont, Burlington, VT 05405 USA; 9 CosmosID, Inc, Rockville, MD 20850 USA; 10 Center for Bioinformatics and Computational Biology, University of Maryland Institute for Advanced Computer Studies (UMIACS), College Park, MD 20742 USA; 11 HudsonAlpha Institute for Biotechnology, Huntsville, AL 35806 USA; 12 Johns Hopkins University Bloomberg School of Public Health, Baltimore, MD USA; 13 Department of Electrical and Computer Engineering, Drexel University, Philadelphia, PA 19104 USA; 14 The Feil Family Brain and Mind Research Institute, New York, NY 10065 USA

**Keywords:** Metagenomics, Shotgun sequencing, Taxonomy, Classification, Comparison, Ensemble methods, Meta-classification, Pathogen detection

## Abstract

**Background:**

One of the main challenges in metagenomics is the identification of microorganisms in clinical and environmental samples. While an extensive and heterogeneous set of computational tools is available to classify microorganisms using whole-genome shotgun sequencing data, comprehensive comparisons of these methods are limited.

**Results:**

In this study, we use the largest-to-date set of laboratory-generated and simulated controls across 846 species to evaluate the performance of 11 metagenomic classifiers. Tools were characterized on the basis of their ability to identify taxa at the genus, species, and strain levels, quantify relative abundances of taxa, and classify individual reads to the species level. Strikingly, the number of species identified by the 11 tools can differ by over three orders of magnitude on the same datasets. Various strategies can ameliorate taxonomic misclassification, including abundance filtering, ensemble approaches, and tool intersection. Nevertheless, these strategies were often insufficient to completely eliminate false positives from environmental samples, which are especially important where they concern medically relevant species. Overall, pairing tools with different classification strategies (k-mer, alignment, marker) can combine their respective advantages.

**Conclusions:**

This study provides positive and negative controls, titrated standards, and a guide for selecting tools for metagenomic analyses by comparing ranges of precision, accuracy, and recall. We show that proper experimental design and analysis parameters can reduce false positives, provide greater resolution of species in complex metagenomic samples, and improve the interpretation of results.

**Electronic supplementary material:**

The online version of this article (doi:10.1186/s13059-017-1299-7) contains supplementary material, which is available to authorized users.

## Background

Sequencing has helped researchers identify microorganisms with roles in such diverse areas as human health [1], the color of lakes [2], and climate [3, 4]. The main objectives when sequencing a metagenomic community are to detect, identify, and describe its component taxa fully and accurately. False positives, false negatives, and speed of analysis are critical concerns, in particular when sequencing is applied to medical diagnosis or tracking infectious agents.

Selective amplification (e.g. 16S, 18S, ITS) of specific gene regions has long been standard for microbial community sequencing, but it introduces bias and omits organisms and functional elements from analysis. Recent large-scale efforts to characterize the human microbiome [5] and a variety of Earth microbiomes [6] used the 16S genes of ribosomal RNA (rRNA) as amplicons. Highly conserved regions within these genes permit the use of common primers for sequencing [7]. Yet certain species of archaea include introns with repetitive regions that interfere with the binding of the most common 16S primers [8, 9] and 16S amplification is unable to capture viral, plasmid, and eukaryotic members of a microbial community [10], which may represent pivotal drivers of an individual infection or epidemic. Moreover, 16S amplification is often insufficient for discrimination at the species and strain levels of classification [11]. Although conserved genes with higher evolutionary rates than 16S rRNA [11] or gene panels could improve discriminatory power among closely related strains of prokaryotes,  these strategies suffer from low adoption and underdeveloped reference databases.

Whole-genome shotgun sequencing addresses some of the issues associated with amplicon-based methods, but other challenges arise. Amplification-based methods remain a cheaper option and 16S databases are more extensive than shotgun databases [12]. Also, taxonomic annotation of short reads produced by most standard sequencing platforms remains problematic, since shorter reads are more likely to map to related taxa that are not actually present in a sample. Classification of whole-genome shotgun data relies on several strategies, including alignment (to all sequences or taxonomically unique markers), composition (*k*-mer analysis), phylogenetics (using models of sequence evolution), assembly, or a combination of these methods. Analysis tools focusing on estimation of abundance tend to use marker genes, which decreases the number of reads classified but increases speed [13]. Tools that classify at the read level have applications beyond taxonomic identification and abundance estimation, such as identifying contaminating reads for removal before genome assembly, calculating coverage, or determining the position of bacterial artificial chromosome clones within chromosomes [14, 15].

Environmental surveys of the New York City (NYC) subway system microbiome and airborne microbes found that metagenomic analysis tools were unable to find a match to any reference genome for about half of input reads, demonstrating the complexity of the data and limitations of current methods and databases [16, 17]. Environmental studies also highlight the importance of reliable species identification when determining pathogenicity. All analysis tools used in the initial NYC subway study detected matches to sequences or markers associated with human pathogens in multiple samples, although subsequent analyses by the original investigators, as well as others, showed there was greater evidence for related, but non-pathogenic, organisms [18–20]. The problem of false positives in metagenomics has been recognized and reported [21, 22]. Strategies including filtering and combining classifiers have been proposed to correct the problem, but a thorough comparison of these strategies has not been done. Recent publications have focused on detecting and identifying harmful or rare microorganisms [20, 22, 23]. However, when studying common non-pathogenic microbes, investigators routinely rely on the accuracy of increasingly rapid analyses from metagenomic classifiers [22].

Fortunately, efforts to standardize protocols for metagenomics, including sample collection, nucleic acid extraction, library preparation, sequencing, and computational analysis are underway, including large-scale efforts like the Microbiome Quality Control (MBQC), the Genome Reference Consortium (GRC), the International Metagenomics and Microbiome Standards Alliance (IMMSA), the Critical Assessment of Metagenomics Interpretation (CAMI), and others [2, 24–28]. Comparisons of available bioinformatics tools have only recently been published [13, 21, 28–30]. For example, Lindgreen, et al. [13] evaluated a set of 14 metagenomics tools, using six datasets comprising more than 400 genera, with the analysis limited to phyla and genera. A similar study by Peabody, et al. [21] evaluated algorithms to the species level but included only two datasets representing 11 species, without taking into account the evolution of the taxonomy of those species [31]. Meanwhile, the number of published tools for the identification of microorganisms continues to increase. At least 80 tools are currently available for 16S and whole-genome sequencing data [32], although some are no longer maintained. Publications describing new methods tend to include comparisons to only a small subset of existing tools, ensuring an enduring challenge in determining which tools should be considered “state-of-the-art” for metagenomics analysis.

To address the challenge, we curated and created a set of 14 laboratory-generated and 21 simulated metagenomic standards datasets comprising 846 species, including read-level and strain-level annotations for a subset of datasets and sequences for a new, commercially available DNA standard that includes bacteria and fungi (Zymo BIOMICS). We further tested tool agreement using a deeply sequenced (>100 M reads) environmental sample and developed new ensemble “voting” methods for improved classification. These data provide an online resource for extant tools and are freely available (http://ftp-private.ncbi.nlm.nih.gov/nist-immsa/IMMSA/) for others to use for benchmarking future tools or new versions of current tools.

## Results

We compared the characteristics and parameters of a set of 11 metagenomic tools [14, 33–44] (Additional file 1: Table S1) representing a variety of classification approaches (*k*-mer composition, alignment, marker). We also present a comprehensive evaluation of their performance, using 35 simulated and biological metagenomes, across a wide range of GC content (14.5–74.8%), size (0.4–13.1 Mb), and species similarity characteristics (Additional file 2: Table S2).

### Genus, species, and subspecies level comparisons

**Fig. 1:**
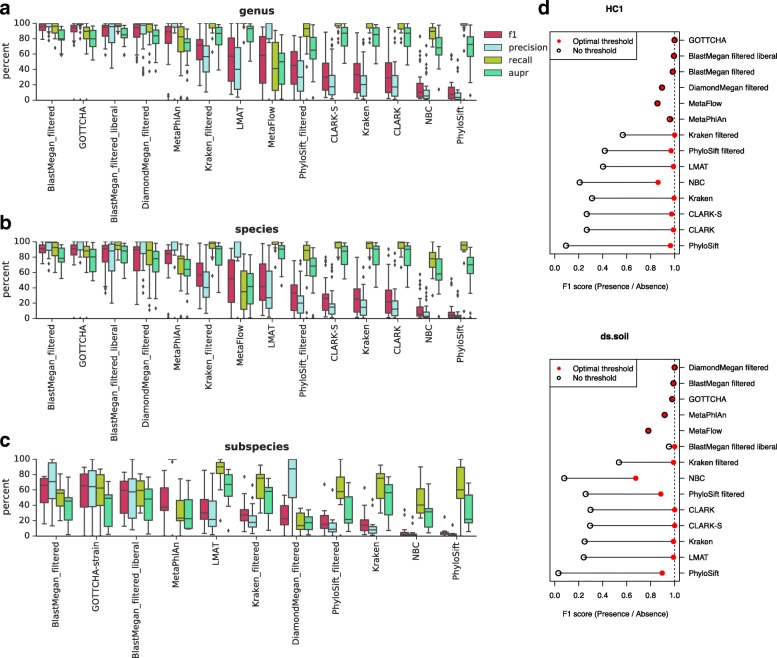
The F1 score, precision, recall, and AUPR (where tools are sorted by decreasing mean F1 score) across datasets with available truth sets for taxonomic classifications at the (**a**) genus (35 datasets), (**b**) species (35 datasets), and (**c**) subspecies (12 datasets) levels. **d** The F1 score changes depending on relative abundance thresholding, as shown for two datasets. The upper bound in *red* marks the optimal abundance threshold to maximize F1 score, adjusted for each dataset and tool. The lower bound in *black* indicates the F1 score for the output without any threshold. Results are sorted by the difference between upper and lower bounds

From the platypus [22] to *Yersinia* *pestis* [17], false positives can plague metagenomic analyses. To evaluate the extent of the problem of false positives with respect to specific tools, we calculated precision, recall, area under the precision-recall curve (AUPR), and F1 score based on detection of the presence or absence of a given genus, species, or subspecies at any abundance. When compared by mean AUPR (mAUPR), all tools performed best at the genus level (45.1% ≤ mAUPR ≤ 86.6%, Fig. 1a), with small decreases in performance at the species level (40.1% ≤ mAUPR ≤ 84.1%, Fig. 1b). Calls at the subspecies (strain) level showed a more marked decrease on all measures for the subset of 12 datasets that included complete strain information (17.3% ≤ mAUPR ≤ 62.5%, Fig. 1c). For *k*-mer-based tools, adding an abundance threshold increased precision and F1 score, which is more affected than AUPR by false positives detected at low abundance, bringing both metrics to the same range for as marker-based tools, which tended to be more precise (Fig. 1d, e).

### Performance across datasets

**Fig. 2:**
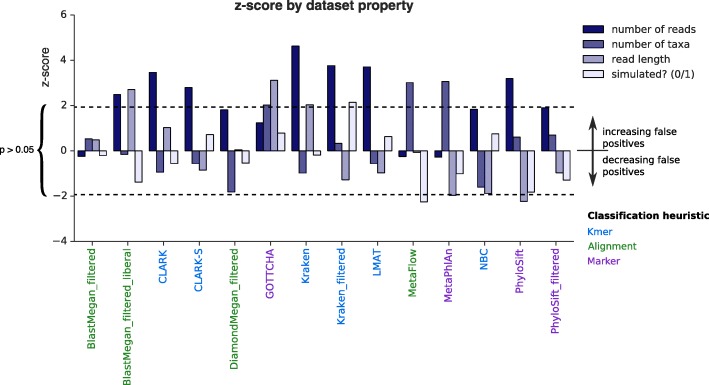
Number of false positives called by different tools as a function of dataset features. The test statistic (z-score) for each feature is reported after fitting a negative binomial model, with *p* value > 0.05 within the *dashed lines* and significant results beyond

Grouping datasets into simulated reads and biological samples revealed that precision is notably lower for biological samples that are titrated and then sequenced (Additional file 3: Figure S1). We initially hypothesized that tools would attain lower precision with biological data because: (1) they detect true contaminants; (2) they detect close variants of the reference strain; or (3) simulated data do not fully capture errors, GC content range, and read distribution biases present in biological data. However, by modeling the number of false positives as a negative binomial of various dataset properties, we found that whether data were simulated had no significant effect on the number of false positives detected for most tools (Fig. 2, with the exception of MetaFlow, which showed a significant trend only with outliers and with few false positives overall, Additional file 3: Figure S2a). The decrease in precision could instead occur because the biological samples contained fewer species on average, but tools detected similar numbers of false positives. No significant relationship was found between the number of taxa in a sample and false positives for most tools. However, false positives for almost all *k*-mer-based methods did tend to increase with more reads (e.g. Additional file 3: Figure S2b), showing a positive relationship between depth and misclassified reads. The same relationship did not exist for most marker-based and alignment-based classifiers, suggesting any additional reads that are miscalled are miscalled as the same species as read depth increases. BLAST-MEGAN and PhyloSift (without or with laxer filters) were exceptions, but adequate filtering was sufficient to avoid the trend. On further examination, the significant relationship between number of taxa and read length and false-positive counts for MetaPhlAn and GOTTCHA appeared weak for MetaPhlAn and entirely due to outliers for GOTTCHA (Additional file 3: Figure S2c–f), indicating misclassification can be very dataset-specific (more below).

The mAUPR for each sample illustrates wide variation among datasets (Additional file 4: Table S3, Additional file 3: Figure S3, Additional file 5: Table S4). Difficulty in identifying taxa was not directly proportional to number of species in the sample, as evidenced by the fact that biological samples containing ten species and simulated datasets containing 25 species with log-normal distributions of abundance were among the most challenging (lowest mAUPR). Indeed, some datasets had a rapid decline in precision as recall increased for almost all tools (e.g. LC5), which illustrates the challenge of calling species with low depth of coverage and the potential for improvement using combined or ensemble methods.

### Ensemble approaches to determine number and identity of species present

**Fig. 3:**
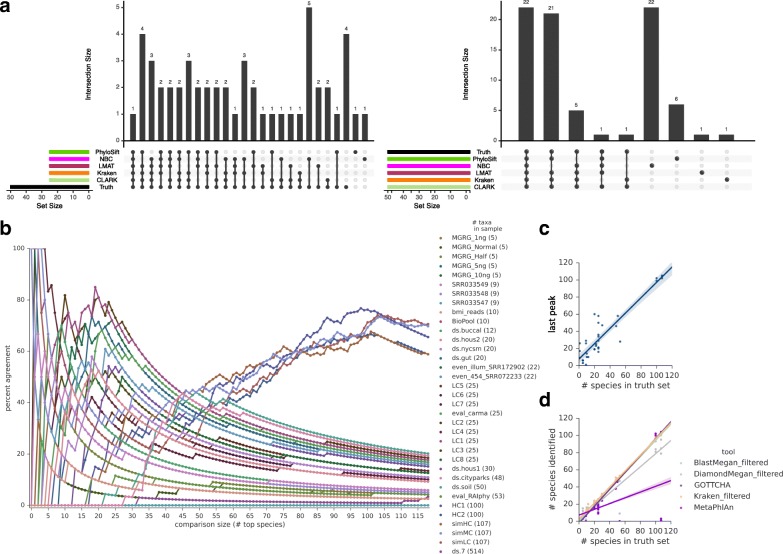
Combining results from imprecise tools can predict the true number of species in a dataset. **a** UpSet plots of the top-X (by abundance) species uniquely found by a classifier or group of classifiers (grouped by *black dots* at bottom, unique overlap sizes in the *bar charts* above). The eval_RAIphy dataset is presented as an example, with comparison sizes X = 25 and X = 50. The percent overlap, calculated as the number of species overlapping between all tools, divided by the number of species in the comparison, increases around the number of species in the sample (50 in this case). **b** The percent overlaps for all datasets show a similar trend. **c** The rightmost peak in (**b**) approximates the number of species in a sample, with a root mean square error (RMSE) of 8.9 on the test datasets. **d** Precise tools can offer comparable or better estimates of species count. RMSE = 3.2, 3.8, 3.9, 12.2, and 32.9 for Kraken filtered, BlastMegan filtered, GOTTCHA, Diamond-MEGAN filtered, and MetaPhlAn2, respectively

To gauge the benefits of combining multiple tools for accuracy and measuring the actual number of species present in a sample, we used a series of tests. First, a combination of five lower-precision tools (CLARK, Kraken, LMAT, NBC, and PhyloSift) showed that the overlap between the most abundant species identified by the tools and the truth set was relatively high for subset sizes close to the actual number of species (Fig. 3a). Concordance among tools was evaluated by sorting species according to abundance and varying the number of results included in the comparison to give a percent $\mathrm{overlap}\kern0.5em =\kern0.5em 100\ast \left(\frac{\#\kern0.5em species\kern0.5em identified\kern0.5em by\kern0.5em all\kern0.5em tools}{\#\kern0.5em species\kern0.5em in\kern0.5em comparision}\right)$ (Fig. 3b). For most samples, discrepancies in results between tools were higher and inconsistent below the known number of species because of differences in abundance estimates. Discrepancies also increased steadily as evaluation size exceeded the actual number of species to encompass more false positives. Thus, these data show that the rightmost peak in percent overlap with even lower-precision tools approximated the known, true number of species (Fig. 3c). However, more precise tools provided a comparable estimate of species number. GOTTCHA and filtered results for Kraken, and BLAST-MEGAN all outperformed the combined-tool strategy for estimating the true number of species in a sample (Fig. 3d).

**Fig. 4:**
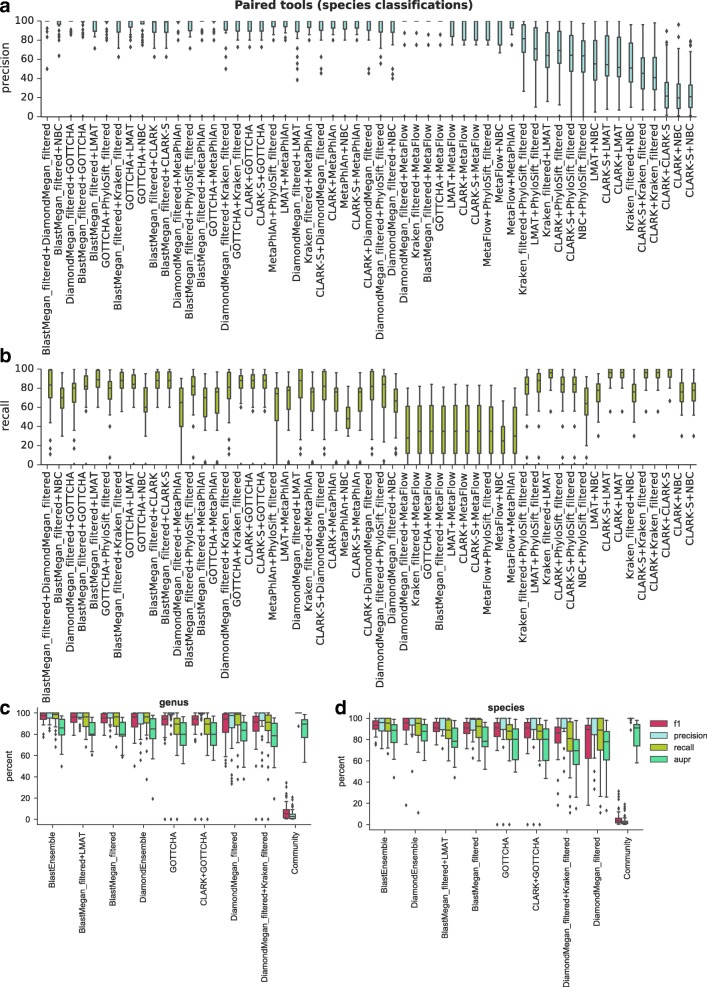
The (**a**) precision and (**b**) recall for intersections of pairs of tools at the species level, sorted by decreasing mean precision. A comparison between multi-tool strategies and combinations at the (**c**) genus and (**d**) species levels. The top unique (non-overlapping) pairs of tools by F1 score from (**a**, **b**) are benchmarked against the top single tools at the species level by F1 score, ensemble classifiers that take the consensus of four or five tools (see “Methods”), and a community predictor that incorporates the results from all 11 tools in the analysis to improve AUPR

Pairwise combinations of tools also show general improvements in taxonomic classification, with the overlap between pairs of tools almost always increasing precision compared to results from individual tools (Fig. 4a). At the species level, combining filtered BLAST-MEGAN with Diamond-MEGAN, NBC, or GOTTCHA, or GOTTCHA with Diamond-MEGAN increased mean precision to over 95%, while 24 other combinations increased precision to over 90%. However, depending on the choice of tools, improvement in precision was incremental at best. For example, combining two *k*-mer-based methods (e.g. CLARK-*S* and NBC, with mean precision 26.5%) did not improve precision to the level of most of the marker-based tools. Increases in precision were offset by decreases in recall (Fig. 4b), notably when tools with small databases such as NBC were added and when tools with different classification strategies (k-mer, alignment, marker) were used.

We next designed a community predictor that combines abundance rankings across all tools (see “Methods”). Consensus ranking offered improvement over individual tools in terms of mAUPR, which gives an idea of the accuracy of abundance rankings (Additional file 5: Table S4). Unlike pairing tools, this approach can also compensate for variations in database completeness among tools for samples of unknown composition, since detection by only a subset of tools was sufficient for inclusion in the filtered results of the community predictor. However, by including every species called by any tool, precision inevitably falls.

As alternatives, we designed two “majority vote” ensemble classifiers using the top tools by F1 score either including BLAST (one of the two slowest tools) or not. At the genus level (Fig. 4c), the majority vote BlastEnsemble had the best F1 score due to limited loss in precision and improved recall. However, we show that little performance is sacrificed using only BLAST-MEGAN or the overlap between BLAST-MEGAN and LMAT. If avoiding BLAST for speed reasons, the majority vote DiamondEnsemble is a competitive alternative, improving the F1 score over Diamond-MEGAN or GOTTCHA alone. At the species level (Fig. 4d), the BlastEnsemble and DiamondEnsemble ranked highest. Finally, pairing tools could occasionally lead to worse performance; for example, GOTTCHA combined with CLARK lowered F1 score compared to GOTTCHA alone (Fig. 4d).

### Classifier performance by taxa

We next sought to identify which species were consistently hardest to detect within and across the tools; the performance of each classifier by taxon is provided in Additional file 6. The most difficult taxa to identify at each taxonomic level (averaged over all classifiers) are *Archaea* (Superkingdom), *Acidobacteria* (phylum), *Acidobacteriia* (class), *Acidobacteriales* (order), *Crocosphaera* (genus), and *Acinetobacter sp. NCTC 10304*/*Corynebacterium pseudogenitalium*/*Propionibacterium sp. 434-HC2* (species). Common phyla such as Proteobacteria, Firmicutes, and Actinobacteria and genera such as *Lactobacillus*, *Staphylococcus*, and *Streptococcus* were frequent false positives. Classifiers show bias towards these taxa likely because they are better represented in databases than others. In terms of false negatives, it is interesting to note that genera that include highly similar species such as *Bacillus*, *Bifidobacterium*, and *Shigella* were commonly miscalled. Species in Additional file 6 are additionally annotated by genomic complexity using the classification groups from Koren, et al. (2014) [45]; however, we found minimal differences between classification groups.

### Negative controls

We tested all tools on a set of three negative controls: sequenced human reference material (NA12878) spiked into a MoBio PowerSoil extraction kit, simulated sequences that do not exist in any species, and environmental samples containing strains previously misclassified as pathogens. Of the methods tested, seven did not include the human genome in their default database. For those that did, human DNA was identified as the most abundant species in the sequencing controls (Additional file 7: Table S5). Most of the tools identified additional non-human species, between a mean of 4.67 for GOTTCHA and 1360 for CLARK-*S*. MetaFlow and BLAST-MEGAN (default filter) were the only tools that did not identify additional species. Notably, not all additional species are necessarily false positives; previous studies (e.g. [46]) detected biological contaminants in sequencing data. Using pairs of tools with mean precision greater than 90% (n = 25) on the test datasets at the genus level, we found *Acinetobacter* and *Escherichia* were genera of putative sequencing and/or reagent contaminants. Previous studies have also detected contamination with both [46]. *Lymphocryptovirus* was also identified by the pairs of tools. High-precision pairs at the species level (n = 28) reported *Escherichia coli, Enterobacter cloacae*, and Epstein-Barr virus. No genera or species were consistently found by pairs of tools with mean precision > 95% (genus n = 15, species n = 4).

We next tested a set of 3 million simulated negative control sequences that do not exist in any known species (see “Methods,” Additional file 2: Table S2). Most tools did not identify any species in these synthetic control sequences, although PhyloSift, NBC, and LMAT identified false positives at low probability scores (PhyloSift) or abundances (NBC and LMAT). The identification of *Sorangium cellulosum* as the most abundant species in all three datasets indicates size bias among NBC’s false positives. The *S. cellulosum* genome is particularly large for bacteria at 13.1 M base pairs [47]. Further top-ranking species from NBC were consistent despite smaller genomes than other organisms in the database, most likely because there are more reference sequences available at the subspecies level for these common microbes (29 *E. coli* and nine *B. cereus* in the NBC database). LMAT consistently identified human as the most abundant species in all three datasets without any other overlap between the datasets, suggesting a bias towards the host reference genome. PhyloSift results were variable, with no species consistently reported in all three datasets.

Finally, we note that filtering is not always sufficient to address the challenge of monophyletic species within certain genera, such as *Bacillus* (Additional file 8: Table S6). In many cases, pairing tools or using ensemble approaches did not reliably correct the problem of species/strain identity, demonstrating that examining plasmids and specific genetic markers is often necessary to correctly characterize pathogenicity, as noted elsewhere [18, 19]. Taxonomic classifiers give a first, useful overview of the sample under investigation but crucial microbes for medically relevant analyses should be validated, visualized, and closely examined, ideally with orthogonal analyses or algorithms. For example, we have released a new tool that can accurately discriminate harmless from pathogenic strains of *Bacillus* using titrated plasmid measures, variant detection, and specific gene markers [20].

### Relative abundance

**Fig. 5:**
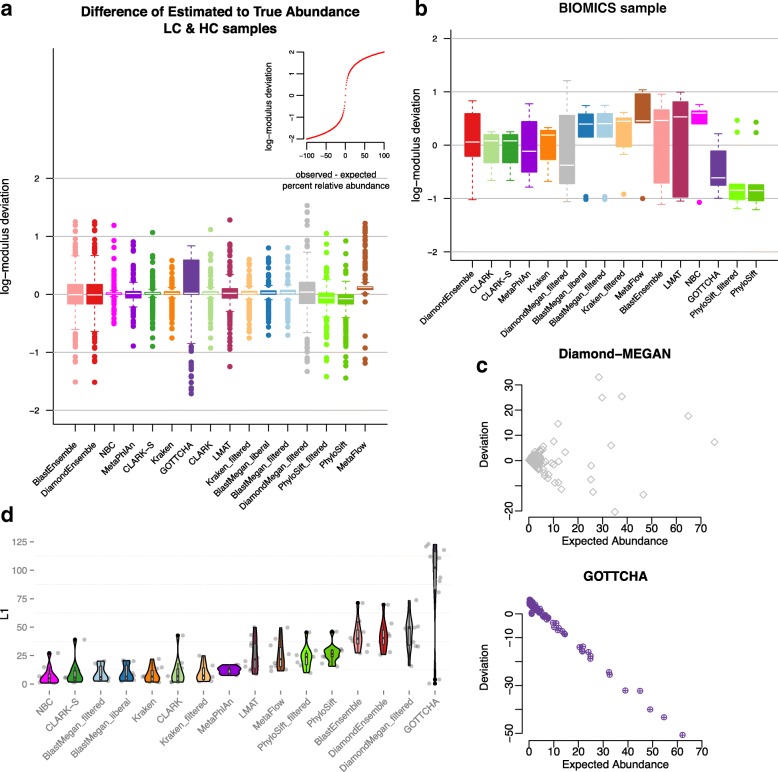
The relative abundances of species detected by tools compared to their known abundances for (**a**) simulated datasets and (**b**) a biological dataset, sorted by median log-modulus difference (difference' = sign(difference)*log(1 + |difference|)). Most differences between observed and expected abundances fell between 0 and 10, with a few exceptions (see *inset* for scale). **c** The deviation between observed and expected abundance by expected percent relative abundance for two high variance tools on the simulated data. While most tools, like Diamond-MEGAN, did not show a pattern in errors, GOTTCHA overestimated low-abundance species and underestimated high-abundance species in the log-normally distributed data. **d** The L1 distances between observed and expected abundances show the consistency of different tools across simulated datasets

After calculating performance based on species detection, we calculated the accuracy of relative abundance predictions (Fig. 5a, b) for titrated and simulated samples. Almost all tools could predict the percentage of a species in a sample to within a few percentage points. GOTTCHA was an exception, performing poorly with log-normally distributed samples (Fig. 5a, c) despite success with more evenly distributed samples (Fig. 5b). Although GOTTCHA showed promise in relative abundance estimation on first publication [29], our results are consistent with those from Lindgreen et al. [13] at higher levels of classification (phylum and genus). While the log-modulus examines a fold-change, the L1 distance shows the distance between relative abundance vectors by dataset (*Σ*_*i* = 1_^*n*^|*y*_*i*_ − *x*_*i*_|), where *y* is the expected profile and *x* the observed profile (Fig. 5d) [48]. Many tools showed greater variation between datasets, as measured by the L1 distance for simulated datasets, especially BLAST and Diamond. The ensemble methods performed the best on the simulated data but had more variation than NBC, MetaPhlAn, and CLARK. On the biological samples, DiamondEnsemble was competitive but again had greater deviation than CLARK and tended to underestimate the relative abundance while CLARK tended to overestimate.

### Limits of detection and depth of sequencing

**Fig. 6:**
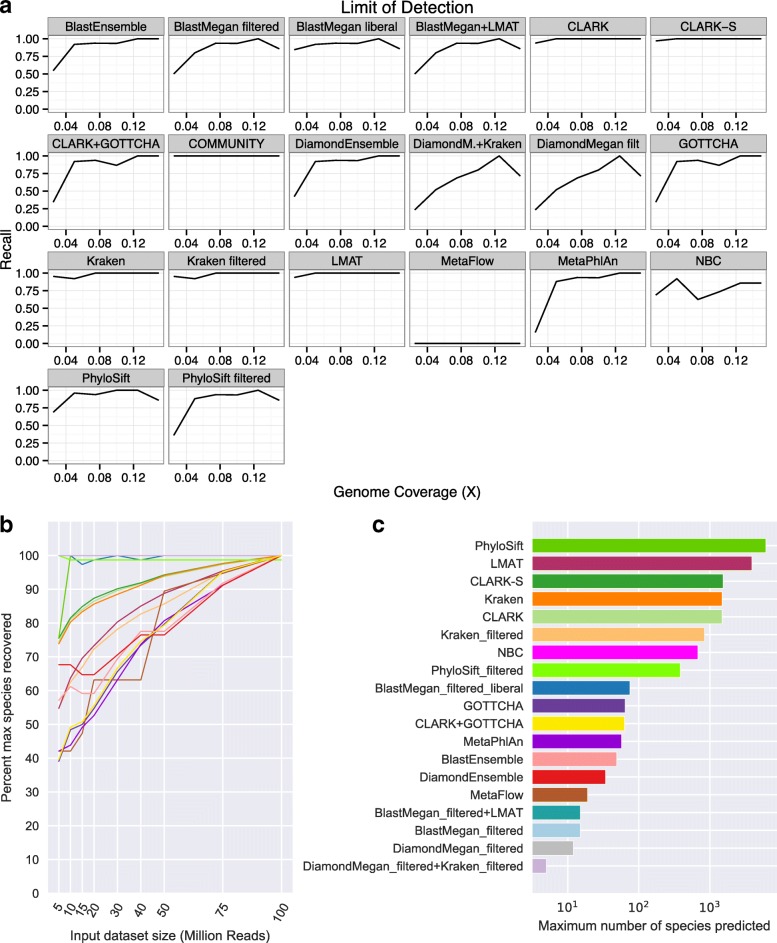
**a** Recall at varying levels of genome coverage on the HC and LC datasets (using the least filtered sets of results for each tool). **b** Downsampling a highly sequenced environmental sample shows depth of sequencing significantly affects results for specific tools, expressed as a percentage of the maximum number of species detected. Depending on strategy, filters can decrease the changes with depth. **c** The maximum number of species detected by each tool at any depth

To quantify the amount of input sequence required for detection, recall was calculated as a function of sequencing depth for each input organism, using the Huttenhower HC/LC datasets (Fig. 6a). Each bin represents 17–69 input organisms, for a total of 197 organisms in the analysis. In general, *k*-mer-based methods (CLARK, Kraken, and LMAT) produced the highest recall, while other methods required higher sequencing depth to achieve equivalent recall.

Yet, sequencing depth can strikingly change the results of a metagenomic study, depending on the tool used. Using a deeply sequenced, complex environmental sample from the New York City subway system (100 M reads from sample P00497), we subsampled the full dataset to identify the depth (5, 10, 15, 20, 30, 40, 50, and 75 M reads) at which each tool recovered its maximum number of predicted species (Fig. 6b). Reinforcing our analysis of limits of detection, marker-based tools identified far more species as depth of sequencing increased, an effect slightly attenuated by filtering (Fig. 6c). Among *k*-mer-based tools, LMAT showed the largest increase, while Kraken, CLARK, and CLARK-*S* showed more gradual increases. Filtering Kraken results decreased the absolute number of species identified but increased the slope of the trend. Notably, only a single species (*Pseudomonas stutzeri*) was called by every method (Additional file 3: Figure S4) and the majority of species called (6223, 72%) were unique to a single tool. Thus, as investigators consider depth of sequencing in their studies, they should keep in mind that results can drastically change, depending on the tool selected and method of filtering. Based on these results, standardizing the sequencing depth and analysis method is extraordinarily important to compare multiple samples within studies or from similar studies.

### Nanopore reads

Short, highly accurate reads are the primary focus of most analysis tools but newer, long-read sequencing methods can offer a lower cost, more portable alternative for metagenomics studies. We tested the tools using two titrated MGRG mixtures (five and 11 species, respectively) sequenced using one of the first available versions (R6 flowcell) and a newer update (R9 flowcell) of the MinION from Oxford Nanopore Technologies (Additional file 3: Figure S5). “2D” consensus-called reads from the initial release of the MinION attained around 80% alignment accuracy, increasing to around 95% since then. Most *k*-mer-based and alignment-based tools identified all component species of the mixture at some level of abundance, although also reported false positives among the top five results. CLARK and Diamond-MEGAN performed as well with lower quality data, while other tools were not as robust. Classification of reads with an average quality score of > Q9 improved results for LMAT. Marker-based methods did not perform well, likely in part because the datasets were small and failed to cover the expected markers.

### Read-level analysis

Finally, we used the output from eight tools that classify individual reads to measure precision and recall for species identification at the read level, where $\mathrm{precision}=\kern0.5em \frac{\#\kern0.5em reads\kern0.5em classified\kern0.5em correctly}{\#\kern0.5em reads\kern0.5em classified}$ and $\mathrm{recall}=\kern0.5em \frac{\#\kern0.5em reads\kern0.5em classified\kern0.5em correctly}{\#\kern0.5em reads}$ with classification to species or subspecies (Additional file 9: Table S7). Both measures were high for all tools, although low recall was observed for some of the datasets, depending on whether the species in the dataset were also in a tool’s database. The low recall of some tools can also be explained by the low proportion of classified reads after filtering (e.g. Diamond-MEGAN and NBC). BLAST-MEGAN offered the highest precision, while CLARK-*S* most frequently provided the highest recall. An ensemble approach was constructed by assigning each read to the most frequently called taxa among the different tools. Setting the quorum to one improved recall by 0.43% on average compared with results from the best single tool for each dataset, while maintaining precision comparable to the most precise tool for each dataset.

### Run-time and memory

**Fig. 7:**
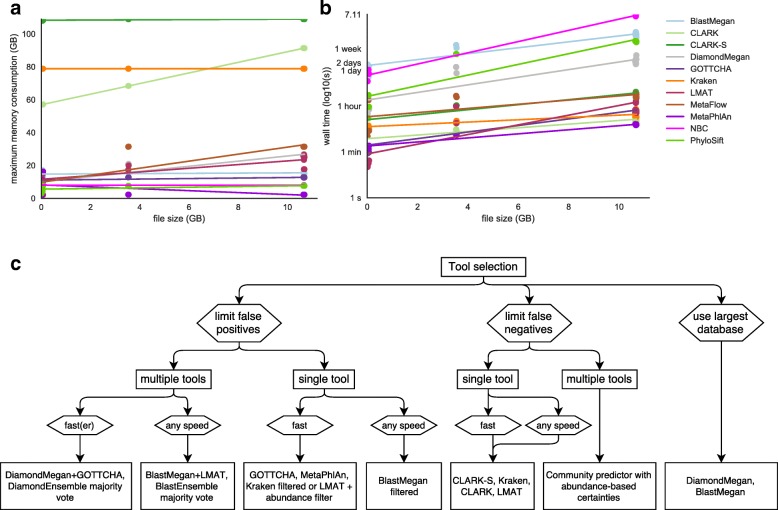
**a** Time and (**b**) maximum memory consumption running the tools on a subset of data using 16 threads (where the option was available, except for PhyloSift, which failed to run using more than one thread, and NBC, which was run through the online server using four threads). BLAST, NBC, and PhyloSift were too slow to completely classify the larger datasets, therefore subsamples were taken and time multiplied. **c** A decision tree summary of recommendations based on the results of this analysis

Speed and memory requirements are often critical factors in the analysis of large-scale datasets. We benchmarked all tools on the same computational cluster, using 16 threads to measure relative speed and memory consumption (Fig. 7). Among the least memory intensive were MetaPhlAn, GOTTCHA, PhyloSift, and NBC. However, PhyloSift was slow compared to CLARK, GOTTCHA, Kraken, MetaFlow, MetaPhlAn, Diamond-Megan and LMAT. NBC and BLAST were the slowest tools, taking multiple weeks to run for larger datasets. Taken together with precision, recall, and database size, these speed constraints can help guide the optimal selection of tools (Fig. 7c).

## Discussion

Recent studies of microbiomes have used a variety of molecular sequencing methods (16S, 18S, ITS, shotgun) to generate data. Many rely on a single classifier or compare the results of a few classifiers, but classifier type and filter use differ among studies [17, 49–53]. To enable greater comparability among metagenome studies, continuous benchmarking on titrated and varied datasets is needed to ensure the accuracy of these tools.

Unlike almost all prior comparisons, our analyses focused on species identification, since species is a taxonomic rank more relevant in clinical diagnostics or pathogen identification than genus or phylum. Although clinical diagnosis and epidemiological tracking often require identification of strains, databases remain poorly populated below the level of species [12, 54]. Classification to strain requires algorithms that can differentiate genomes and their plasmids with high similarity, as we have shown for *Bacillus*, which is particularly challenging when using short reads. Most of the test datasets included in this study lacked complete information at the strain level, so we were able to calculate precision and recall for only a subset of datasets (n = 12). These results clearly indicate that specialized approaches are still needed. For example, PanPhlAn [55] and MetaPhlAn2 strainer are recent tools designed by the authors of MetaPhlAn for epidemiological strain detection, although they focus on relationships between strains in a sample for a given species, rather than strain identification of all species in a sample. ConStrains [56] instead uses single nucleotide polymorphism profiling and requires higher depth of coverage than available for the datasets used in this study.

Every database ideally should provide a complete set of taxa for sequence comparison. In reality, most species lack reference genomes, with contigs or full genomes for only around 300,000 microbial species of a recent estimate of up to 1 trillion extant species globally [57]. Large databases also demand greater computational resources, another reason that tools classify samples using limited sets of reference genomes. However, incomplete databases result in more unclassified reads or incorrect identification of reads as related species. For this study, tools were compared using their default or recommended databases, where possible. Thus, our analyses penalize tools if their databases are missing genera or species in the truth set for a sample. We considered this a fair comparison since database size can affect the results of metagenomic analyses significantly (as we demonstrate with the limited NBC database) and certain tools were trained on, or provide, a single database.

By considering tools in their entirety, this study does not directly address differences between databases, but in the absence of any other guide for specific problems, users of these tools usually choose the default or most readily available database. Differences between tools’ default databases are shown in Additional file 1: Table S1. For example, for full metagenomic profiling across all kingdoms of life, BLAST and Diamond offer the most extensive databases for eukaryotes, although databases can be constructed for tools like CLARK or Kraken to include greater kingdom diversity. One issue we note is that results for web-based tools that frequently update their databases (e.g. BLAST) vary over time, and may not be reproducible between analyses. The high percentage of unidentifiable reads, or “microbial dark matter,” in many studies [16, 17] underscores the limitations of databases currently available, as well the use for de novo assembly of reads to help with the uncharacterized microorganisms from the field.

Long read technologies, such as the MinION nanopore, 10X Genomics, or PacBio sequencers can be helpful both for de novo assembly [58, 59] and avoiding ambiguous mapping of reads from conserved regions. Our results suggest that even relatively low-quality reads (below an average base quality of 9) can be used for taxonomic classification, with improvements as dataset size and quality increased. Most *k*-mer-based and alignment-based methods performed well with longer reads, while marker-based tools did not.

## Conclusions

These data and results provide useful metrics, datasets (positive and negative controls), and best practices for other investigators to use, including well-characterized, titrated reference datasets now routinely sequenced by laboratories globally. Using the simulated datasets, read-level accuracy can be calculated and aid in determining the role of read ambiguity in taxonomic identification. Our data showed that read-level precision was much higher than organism-level precision for some tools, including CLARK, Kraken, and NBC. By varying the filtering threshold for identification and comparing F1 scores to AUPR, we showed that the discrepancy occurs because these tools detect many taxa at relatively low read counts.

To determine which taxa are actually present in a sample, users can filter their results to increase precision and exercise caution in reporting detection of low abundance species, which can be problematic to call. For example, an analysis of environmental samples collected in the Boston subway system filtered out organisms present at less than 0.1% of total abundance and in fewer than two samples [60]. Yet, depending on tool selection, this filter would have been insufficient to reject strains of *Bacillus* in the NYC subway study, despite the absence of pathogenic plasmids that distinguish it from closely related species [17]. Therefore, filters must be considered in the context of a given study along with additional information like plasmids, genome coverage, markers’ genetic variants, presence of related species, and epidemiology. Filters should be used with consideration for study design and read depth, as well as the classification tool used. Nevertheless, discarding all taxa at low abundance risks rejecting species that are actually present. For instance, highly complex microbial communities found in the adult human gut and in soil contain species numbering in the hundreds and tens of thousands, respectively [61, 62]. Assuming even abundance and depth of coverage, any one species would be represented by less than 0.1% of reads. In a real community of variable species abundance, many species would compose an even smaller percentage [51].

There are several options to address the ongoing problem of thresholds and low abundance species. First, precision–recall curves using known samples (such as those used in this study) can help define the appropriate filtering threshold for a given tool. Second, combining predictions from several tools offers an alternative means to improve species detection and multiple ensemble approaches were explored in this study. Finally, targeted methods (e.g. capture, polymerase chain reaction, direct hybridization) can confirm the presence of rare taxa or specific pathogens. As citizen science expands with cheaper and more accessible sequencing technologies [63, 64], it is important that background on bioinformatics tools is provided, that classifier results are not oversold, and that genus-level differences are viewed as trends, not diagnostics.

Although many approaches are possible, here we explored ensemble methods without taking into account the differences in performance of their component tools to avoid overfitting weighted schemes. Trained predictors merit further research, including variations on that recently proposed by Metwally, et al. [65]. Any ensemble method requires combining outputs of various tools, a challenge that would benefit by the adoption of standardized file formats. The Critical Assessment of Metagenomic Interpretation challenge proposed one such unifying format [27]. Inclusion of NCBI taxonomy IDs in addition to taxa names, which are more variable and difficult to track across database updates, would greatly simplify comparisons.

With significant variation in tools’ performance demonstrated in this study, continual benchmarking using the latest sequencing methods and chemistries is critical. Tool parameters, databases, and test dataset features all affect the measures used for the comparisons. Benchmarking studies need to be computationally reproducible and transparent and use readily available samples and methods. We showed here that filtering and combining tools decreases false positives, but that a range of issues still affect the classification of environmental samples, including depth of sequencing, sample complexity, and sequencing contamination. Additional benchmarking is necessary for analyses such as antibiotic resistance marker identification, functional classification, and mobile genetic elements; this is especially important as metagenomics moves towards answering fundamental questions of cross-kingdom genetic dynamics. Metrics of tool performance can inform the implementation of tools across metagenomics research studies, citizen science, and “precision metagenomics,” where robust metagenomics analysis can guide clinical decisions across all kingdoms of life.

## Methods

### Data selection

A wide range of datasets was selected to answer a variety of questions. Published datasets with known species compositions (“truth sets,” see Additional file 2: Table S2) were chosen to measure precision and recall. Additional datasets with known abundances, including a subset with even (HC datasets) and log-normal (LC datasets) distributions of species, facilitated analysis of abundance predictions and limits of detection. The MGRG libraries sequenced using Illumina and the MinION nanopore sequencer contain equimolar concentrations of DNA from five organisms.

We used two sets of negative controls: biological controls to test for contamination during sample preparation; and a simulated set of reads that did not map to any known organisms to test for spurious predictions. The biological control was made by spiking human NA12878 samples into a MoBio PowerSoil kit and then extracting and sequencing the DNA in triplicate. The three simulated negative control datasets we use include 100-bp reads constructed from 17-mers that do not map to any genomes in the full NCBI/RefSeq database [37].

Lack of agreement in read classification among the tools, which can arise from discrepancies in the databases, classification algorithms, and underlying read ambiguity, was investigated. Notably, 100-bp reads are short enough that some will map to several distinct organisms (e.g. from the same genus) within a given error rate. To facilitate a comparison between tools based solely on the database of the tool and internal sequence analysis algorithm, datasets of reads that map unambiguously to a single species within the NCBI/RefSeq database were generated using a methodology described previously [37]. Briefly, six datasets were created using the ART simulator with default error and quality base profiles [66] to simulate 100-bp Illumina reads from sets of reference sequences at a coverage of 30X and efficiently post-processed to remove ambiguously mapped read at the species levels [36]. Each of these unambiguous datasets (“Buc12,” “CParMed48,” “Gut20,” “Hou31,” “Hou21,” and “Soi50”) represents a distinct microbial habitat based on studies that characterized real metagenomes found in the human body (mouth, gut, etc.) and in the natural or built environment (city parks/medians, houses, and soil), while a seventh dataset, “simBA-525,” comprised 525 randomly selected species. An extra unambiguous dataset, “NYCSM20,” was created to represent the organisms of the New York City subway system as described in the study of Afshinnekoo et al. [17], using the same methodology as in Ounit and Lonardi [37]. Together, these eight unambiguous datasets contain a total of 657 species. In the survey of the NYC subway metagenome, Afshinnekoo et al. noted that two samples (P00134 and P00497) showed reads that mapped to *Bacillus anthracis* using MetaPhlAn2, SURPI, and MegaBLAST-MEGAN, but it has been since shown by the authors and others that this species identification was incorrect. We used the same datasets to test for the detection of a pathogenic false positive using the wider array of tools included in this study [20].

### Tool commands

#### CLARK series

We ran CLARK and CLARK-*S*. CLARK is up to two orders of magnitude faster than CLARK-*S* but the latter is capable of assigning more reads with higher accuracy at the phylum/genus level [67] and species level [37]. Both were run using databases built from the NCBI/RefSeq bacterial, archaeal, and viral genomes.

CLARK was run on a single node using the following commands:

- $./set_target.sh < DIR > bacteria viruses *(to set the databases at the species level)*
- $./classify_metagenome.sh -O < file > .fasta -R < result > *(to run the classification on the file named < file > .fasta given the database defined earlier)*
- $./estimate_abundance -D < DIR > -F result.csv > result.report.txt *(to get the abundance estimation report)*

CLARK-*S* was run on 16 nodes using the following commands:

- $./set_target.sh < DIR > bacteria viruses
- $./buildSpacedDB.sh *(to build the database of spaced 31-mers, using three different seeds)*
- $./classify_metagenome.sh -O < file > -R < result > -n 16 --spaced
- $./estimate_abundance -D < DIR > -F result.csv -c 0.75 -g 0.08 > result.report.txt

For CLARK-*S*, distribution plots of assignments per confidence or gamma score show an inconsistent peak localized around low values likely due to sequencing errors or noise, which suggests 1–3% of assignments are random or lack sufficient evidence. The final abundance report was therefore filtered for confidence scores ≥ 0.75 (“-c 0.75”) and gamma scores ≥ 0.08 (“-g 0.08”).

We note that we used parameters to generate classifications to the level of species for all analyses, although classifying only to genus could improve results at that level. Speed measurements were extracted from the log.out files produced for each run.

#### GOTTCHA

Since GOTTCHA does not accept input in fasta format, fasta files for simulated datasets were converted to fastqs by setting all base quality scores to the maximum.

The v20150825 bacterial databases (GOTTCHA_BACTERIA_c4937_k24_u30_xHUMAN3x.strain.tar.gz for the strain-level analyses and GOTTCHA_BACTERIA_c4937_k24_u30_xHUMAN3x.species.tar.gz for all others) were then downloaded and unpacked and GOTTCHA run using the command:

- $ gottcha.pl --threads 16 --outdir $TMPDIR/--input $TMPDIR/$DATASET.fastq --database $DATABASE_LOCATION

As for CLARK and CLARK-*S*, using the genus databases for classifications to genus could improve results at that level (although we observed only small differences in our comparisons to use of the species databases for a few datasets).

#### Kraken

Genomes were downloaded and a database built using the following commands:

- $ kraken-build --download-taxonomy --db KrakenDB
- $ kraken-build --download-library bacteria --db KrakenDB
- $ kraken-build --build --db KrakenDB --threads 30
- $ clean_db.sh KrakenDB

Finally, Kraken was run on fasta and fastq input files using 30 nodes (or 16 for time/memory comparisons).

- $ time kraken --db < KrakenDB > --threads 30 --fast[a/q]-input [input file] > [unfiltered output]

Results were filtered by scores for each read (# of *k*-mers mapped to a taxon/# of *k*-mers without an ambiguous nucleotide) using a threshold of 0.2, which had been shown to provide a per-read precision of ~99.1 and sensitivity ~72.8 (http://ccb.jhu.edu/software/kraken/MANUAL.html).

- $ time kraken-filter --db < KrakenDB > --threshold 0.2 [unfiltered output] > [filtered output]

Both filtered and unfiltered reports were generated using

- $ kraken-report --db < KrakenDB > [filtered/unfiltered output] > [report]

Paired end files were run with the --paired flag.

We compared results using the standard database and the “mini” database of 4 GB, which relies on a reduced representation of *k*-mers. Precision, recall, F1 score, and AUPR were highly similar; therefore, we show only the results for the full database.

#### LMAT

We used the larger of the available databases, lmat-4-14.20mer.db, with the command

- $ run_rl.sh --db_file=/dimmap/lmat-4-14.20mer.db --query_file = $file --threads = 96 --odir = $dir --overwrite

#### MEGAN

- BLAST
  We downloaded the NCBI BLAST executable (v2.2.28) and NT database (nucleotide) from ftp://ftp.ncbi.nlm.nih.gov/blast/. We searched for each unpaired read in the NT database using the Megablast mode of operation and an e-value threshold of 1e-20. The following command appended taxonomy columns to the standard tabular output format:
  - $ blastn –query < sample > .fasta -task megablast -db NT -evalue 1e-20 \
  -outfmt '6 std staxids scomnames sscinames sskingdoms'" \
  - < sample > .blast
  We downloaded and ran MEGAN (v5.10.6) from http://ab.inf.uni-tuebingen.de/software/megan5/. We ran MEGAN in non-interactive (command line) mode as follows:
  - $ MEGAN/tools/blast2lca --format BlastTAB –topPercent 10 \
  --input < sample > .blast --output < sample > _read_assignments.txt
  This MEGAN command returns the lowest common ancestor (LCA) taxon in the NCBI Taxonomy for each read. The topPercent option (default value 10) discards any hit with a bitscore less than 10% of the best hit for that read.
  We used a custom Ruby script, summarize_megan_taxonomy_file.rb, to sum the per-read assignments into cumulative sums for each taxon. The script enforced the MEGAN parameter, Min Support Percent = 0.1, which requires that at least this many reads (as a percent of the total reads with hits) be assigned to a taxon for it to be reported. Taxa with fewer reads are assigned to the parent in the hierarchy. Output files were given the suffix “BlastMeganFiltered” to indicate that an abundance threshold (also called a filter in this manuscript) was applied. We produced a second set of output files using 0.01 as the minimum percentage and named with the suffix “BlastMeganFilteredLiberal.”
- DIAMOND
  DIAMOND (v0.7.9.58) was run using the nr database downloaded on 2015-11-20 from NCBI (ftp://ftp.ncbi.nih.gov/blast/db/FASTA/). We tried both normal and --sensitive mode, with very similar results and present the results for the normal mode. The command to execute DIAMOND with input file sample_name.fasta is as follows and generates an output file named sample_name.daa
  diamond blastx -d/path/to/NCBI_nr/nr -q sample_name.fasta -a sample_name -p 16
  MEGAN (v5.10.6) (obtained as described above) was used for read-level taxonomic classification in non-interactive mode:
  - megan/tools/blast2lca --input sample_name.daa --format BlastTAB --topPercent 10 --gi2taxa megan/GI_Tax_mapping/gi_taxid-March2015X.bin --output sample_name.read_assignments.txt
  A custom Ruby script (described above) was used to sum the per-read assignments into cumulative sums for each taxon.

#### MetaFlow

MetaFlow is an alignment-based program using BLAST for fasta files produced by Illumina or 454 pyrosequencing (all fastqs for this study were converted to fastas to run MetaFlow). Any biological sample that was not sequenced with one of these technologies was not run or analyzed by MetaFlow. We ran MetaFlow using the recommended parameters as described in the available tutorial (https://github.com/alexandrutomescu/metaflow/blob/master/TUTORIAL.md). We first installed the default microbial database from NBCI/RefSeq and built the associated BLAST database. Using the provided script “Create_Blast_DB.py,” the genomes are downloaded and stored in the directory “NCBI” in the working directory and the BLAST database is created with the command:

- $ makeblastdb -in NCBI_DB/BLAST_DB.fasta -out NCBI_DB/BLAST_DB.fasta -dbtype nucl

Classification of each sample (<sample > .fasta) then proceeded through the following steps:

1. BLAST alignment
  $ blastn -query < sampleID > .fasta -out < sampleID > .blast -outfmt 6 -db NCBI_DB/BLAST_DB.fasta -num_threads 10
  We converted the sample file into FASTA file if the sample file was in FASTQ format and used the default settings to align the reads with BLAST.
2. LGF file construction
  $ python BLAST_TO_LGF.py < sampleID > .blast NCBI_DB/NCBI_Ref_Genome.txt < avg_length > <seq_type > 
  The graph-based representation from the BLAST alignments is built into a LGF (Lemon Graph Format) file. This operation takes as input the average length (<avg_length>) of the reads and the sequencing machine (<seq_type>, 0 for Illumina and 1 for 454 pyrosequencing).
3. MetaFlow
  $./metaflow -m < sampleID > .blast.lgf -g NCBI_DB/NCBI_Ref_Genome.txt -c metaflow.config
  The MetaFlow program is finally run using as input the LGF file (from the previous step), the database metadata (i.e. genome length) and a configuration file. We used the default settings for the configuration but lowered the minimum threshold for abundance to increase the number of detected organisms from 0.3 to 0.001). The program outputs all the detected organisms with their related abundance and relative abundance.

#### MetaPhlAn2

MetaPhlAn2 was run using suggested command under “Basic usage” with the provided database (v20) and the latest version of bowtie2 (bowtie2-2.2.6):

- $ metaphlan2.py metagenome.fasta --mpa_pkl ${mpa_dir}/db_v20/mpa_v20_m200.pkl --bowtie2db ${mpa_dir}/db_v20/mpa_v20_m200 --input_type fasta > profiled_metagenome.txt

#### NBC

All datasets were analyzed through the web interface using the original bacterial databases [42], but not the fungal/viral or other databases [68].

Results were further filtered for the read-level analysis because every read is classified by default, using a threshold = -23.7*Read_length + 490 (suggested by http://nbc.ece.drexel.edu/FAQ.php).

#### PhyloSift

PhyloSift was run using

- $ phylosift all [--paired] < fasta or fastq > .gz

Results were filtered for assignments with > 90% confidence.

### Analysis

#### Taxonomy IDs

For those tools that do not provide taxonomy IDs, taxa names were converted using the best matches to NCBI names before comparison of results to other tools and truth sets. A conversion table is provided in the supplementary materials (Additional file 10).

#### Precision–recall

Precision was calculated as $\frac{\#\kern0.5em species\kern0.5em identified\kern0.5em correctly}{\#\kern0.5em species\kern0.5em identified}$ and recall as $\frac{\#\kern0.5em species\kern0.5em identified\kern0.5em correctly}{\#\kern0.5em species\kern0.5em in\kern0.5em the\kern0.5em truth\kern0.5em set}$. We calculated precision–recall curves by successively filtering out results based on abundances to increase precision and recalculating recall at each step, defining true and false positives in terms of the binary detection of species. The AUPR was calculated using the lower trapezoid method [69]. For subspecies, classification at varying levels complicated the analysis (e.g. *Salmonella enterica* subsp. enterica, *Salmonella enterica* subsp. enterica serovar Typhimurium, *Salmonella enterica* subsp. enterica serovar Typhimurium str. LT2). We accorded partial credit if higher levels of subspecies classification were correct but the lowest were not by expanding the truth sets to include all intermediate nodes below species.

#### Negative binomial model

Negative binomial regression was used to estimate the contributions of dataset features to the number of false positives called by each tool. Using all 40 datasets, the false-positive rate was modeled as false positives ~ ß0 + ß1(X1) + ß2(X2) + ß3(X3) + ß4(X4), where X = (number of reads, number of taxa, read length, and a binary variable indicating whether a dataset is simulated). Test statistics and associated *p* values were calculated for each variable using the glm.nb function in R.

#### Abundance

Abundances were compared to truth set values for simulated and laboratory-sequenced data. Separate truth sets were prepared for comparison to tools that do and do not provide relative abundances by scaling expected relative abundances by genome size and ploidy (expected read proportion = (expected relative abundance)/(genome length*ploidy)) or comparing directly to read proportions. The genome size and ploidy information were obtained from the manual for the BIOMICS™ Microbial Community DNA Standard, while the read proportions for the HC and LC samples were calculated using species information from the fasta file headers. The log-modulus was calculated as y' = sign(y)*log10(1 + |y|) to preserve the sign of the difference between estimated and expected abundance, y.

#### Community/ensemble predictors

Ensemble predictors were designed to incorporate the results from multiple tools using either summaries of identified taxa and/or their relative abundances, or read-level classifications.

#### Summary-based ensembles

##### Community

When multiple tools agree on inferred taxa, it increases confidence in the result. Conversely, when multiple tools disagree on inferred taxa, it diminishes confidence in the result. To study this intuition quantitatively, we formulated a simple algorithm for combining the outputs from multiple tools into a single “community” output. For each tool, we first ranked the taxa from largest to smallest relative abundance, such that the most abundant taxon is rank 1 and the least abundant taxon is rank n. Next, we weighted taxa by 1/rank, such that the most abundant taxon has a weight 1 and the least abundant taxon has weight 1/n. Finally, we summed the weights for each taxon across the tools to give the total community weight for each taxon. For example, if *E. coli* were ranked second by five of five tools, the total weight of *E. coli* would be 5/2. Variations on this method of combining multiple ranked lists into a single list have been shown to effectively mitigate the uncertainty about which tool(s) are the most accurate on a particular dataset [70, 71] and for complex samples [72].

##### Quorum

As an alternative approach, we tested various combinations of three to five classifiers to predict taxa present based on the majority vote of the ensemble (known as majority-vote ensemble classifiers in machine learning literature). In the end, tools with the highest precision/recall (BlastMEGAN_Filtered, GOTTCHA, DiamondMEGAN_Filtered, Metaphlan, Kraken_Filtered, and LMAT) were combined to yield the best majority vote combinations. We limited the ensembles to a maximum of five classifiers, reasoning that any performance gains with more classifiers would not be worth the added computational time. Two majority vote combinations were chosen: (1) BlastEnsemble, a majority vote classifier that relies on one of the BLAST-based configurations, with a taxa being called if two or more of the classifiers call it out of the calls from BlastMEGAN (filtered), GOTTCHA, LMAT, and MetaPhlAn; and (2) DiamondEnsemble, a majority vote classifier that does not rely on BLAST, with three or more of Diamond-MEGAN, GOTTCHA, Kraken (filtered), LMAT, and MetaPhlAn calling a taxa. The second was designed to perform well but avoid BLAST-MEGAN, the tool with the highest F1 score but also one of the slowest tools.

In order to get the final relative abundance value, we tried various methods, including taking the mean or median of the ensemble. We settled on a method that prioritizes the classifiers based on L1 distance for the simulated data. Therefore, in the BlastEnsemble, the BLAST-MEGAN relative abundance values were taken for all taxa that were called by BLAST-MEGAN and the ensemble, then MetaPhlAn abundance values were taken for taxa called by the BlastEnsemble but not BLAST, then LMAT values were taken for taxa called by LMAT and the ensemble but not BLAST or MetaPhlAn, and finally GOTTCHA values. This method was also applied to the DiamondEnsemble, with Kraken (filtered) prioritized, followed by MetaPhlAn, LMAT, Diamond, and GOTTCHA. To compensate for any probability mass loss, the final relative abundance values (numerator) were divided by the sum of the relative abundance after excluding any taxa not called by the ensembles (denominator).

#### Read-based ensembles

For each read *r* of a given dataset, this predictor considers the classification results given by all the tools and classifies *r* using the majority vote and a “quorum” value (set in input). If all the tools agree on the assignment of *r*, say organism *o*, then the predictor classifies *r* to *o* and moves to the next read, otherwise the predictor identifies the organism *o’* of the highest vote count *v* and classifies *r* to *o’* if *v* is higher than a quorum value set by the user (ties are broken arbitrarily).

Parameters are the results of the tools (i.e. a list of pairs containing the read identifiers and the associated organism predicted) and a quorum value (e.g. 1, 2, … 7). Note that we have set the predictor to ignore cases in which only one tool provides a prediction.

#### Time/Memory profiling

We profiled the time and memory consumption of the tools using the “/usr/bin/time” command on the same Linux cluster at Weill Cornell. PhyloSift failed to run without error using multiple threads; otherwise we ran tools using 16 threads when given an option. Wall time and maximum resident set size are presented in Fig. 7. NBC finished running on only a subset of samples, while we had to subdivide larger files to run BLAST and PhyloSift to completion. The overall maximum memory and cumulative time (with extrapolations from the subsampled files where only a subset finished running) were taken as estimates in these cases.

## Additional files

Additional file 1: Table S1. Comparison of tools by classification strategies and associated databases. (XLS 49 kb)

Additional file 2: Table S2. Features of datasets included in the analysis. Mean AUPR across tools provides an indication of the difficulty of a dataset. (XLSX 18 kb)

Additional file 3: Supplementary figures. (PDF 496 kb)

Additional file 4: Table S3. Precision and recall at the species level for tool, listed by dataset. (XLSX 52 kb)

Additional file 5: Table S4. Mean and median AUPR for the community predictor vs. other tools. (XLSX 44 kb)

Additional file 6: Tool accuracy per taxon. Each file is categorized by taxonomic level. Inside each file, the first sheet shows the accuracy (with additional columns at the species level for mean GC content, genome length, and class(es) for associated strains based on the number of repeats). The second sheet details the number of false positives and the third sheet details the number of false negatives of each classifier for each taxon in each taxonomic level. The three ensemble classifiers (Community, Blast Ensemble, Diamond Ensemble) are included in this analysis for comparison. (ZIP 1590 kb)

Additional file 7: Table S5. Negative control results across tools for sequencing blanks with human DNA spiked in and simulated data constructed from nullomers (17-mers that do not map to any reference). (XLSX 39 kb)

Additional file 8: Table S6. The read counts and relative abundances for *Bacillus anthracis* identified by various tools after the whole genome sequencing of two samples from the New York City subway system. (XLSX 13 kb)

Additional file 9: Table S7. Read-level analysis of 21 datasets for seven classifiers and two meta-classifiers that aim to maximize precision and recall, respectively. (DOCX 33 kb)

Additional file 10: Name to taxonomy ID conversion tables for tools that do not report taxonomy IDs. (ZIP 220 kb)
