# Correction to: Comprehensive benchmarking and ensemble approaches for metagenomic classifiers

**DOI:** 10.1186/s13059-019-1687-2

**Published:** 2019-04-05

**Authors:** Alexa B. R. McIntyre, Rachid Ounit, Ebrahim Afshinnekoo, Robert J. Prill, Elizabeth Hénaff, Noah Alexander, Samuel S. Minot, David Danko, Jonathan Foox, Sofia Ahsanuddin, Scott Tighe, Nur A. Hasan, Poorani Subramanian, Kelly Moffat, Shawn Levy, Stefano Lonardi, Nick Greenfield, Rita R. Colwell, Gail L. Rosen, Christopher E. Mason

**Affiliations:** 1 Tri-Institutional Program in Computational Biology and Medicine, New York, NY USA; 2 Department of Physiology and Biophysics, Weill Cornell Medicine, New York, NY 10021 USA; 3 The HRH Prince Alwaleed Bin Talal Bin Abdulaziz Alsaud Institute for Computational Biomedicine, New York, NY 10021 USA; 4 Department of Computer Science and Engineering, University of California, Riverside, CA 92521 USA; 5 School of Medicine, New York Medical College, Valhalla, NY 10595 USA; 6 Accelerated Discovery Lab, IBM Almaden Research Center, San Jose, CA 95120 USA; 7 One Codex, Reference Genomics, San Francisco, CA 94103 USA; 8 University of Vermont, Burlington, VT 05405 USA; 9 CosmosID, Inc, Rockville, MD 20850 USA; 10 Center for Bioinformatics and Computational Biology, University of Maryland Institute for Advanced Computer Studies (UMIACS), College Park, MD 20742 USA; 11 HudsonAlpha Institute for Biotechnology, Huntsville, AL 35806 USA; 12 Johns Hopkins University Bloomberg School of Public Health, Baltimore, MD USA; 13 Department of Electrical and Computer Engineering, Drexel University, Philadelphia, PA 19104 USA; 14 The Feil Family Brain and Mind Research Institute, New York, NY 10065 USA

**Correction to: Genome Biol (2017) 18:182**

**https://doi.org/10.1186/s13059-017-1299-7**

Following publication of the original article [1], the authors would like to highlight the following two corrections:

1. The updated “Availability of data and materials” declaration to the article:
  The datasets and scripts supporting the conclusions of this article are freely and publicly available through the IMMSA server, ftp://ftp-private.ncbi.nlm.nih.gov/nist-immsa/IMMSA/
  Scripts used for analysis and generating figures are available at: https://scu.med.cornell.edu/git/abm237/benchmarking_metagenomic_classifiers
2. The authors would like to clarify that the kraken db build command in the manuscript is for the bacterial database; the command for the standard db is available through the kraken manual: kraken-build --standard --db $DBNAME
