# Supplementary material for: Comprehensive benchmarking and ensemble approaches for metagenomic classifiers
Source: Genome Biol. 2017 Sep 21;18:182. doi: 10.1186/s13059-017-1299-7 (PMC5609029; doi:10.1186/s13059-017-1299-7)
Supplement: Supplementary file 3 — Supplementary figures. (PDF 496 kb) [file 13059_2017_1299_MOESM3_ESM.pdf]

Supplementary Figure 1

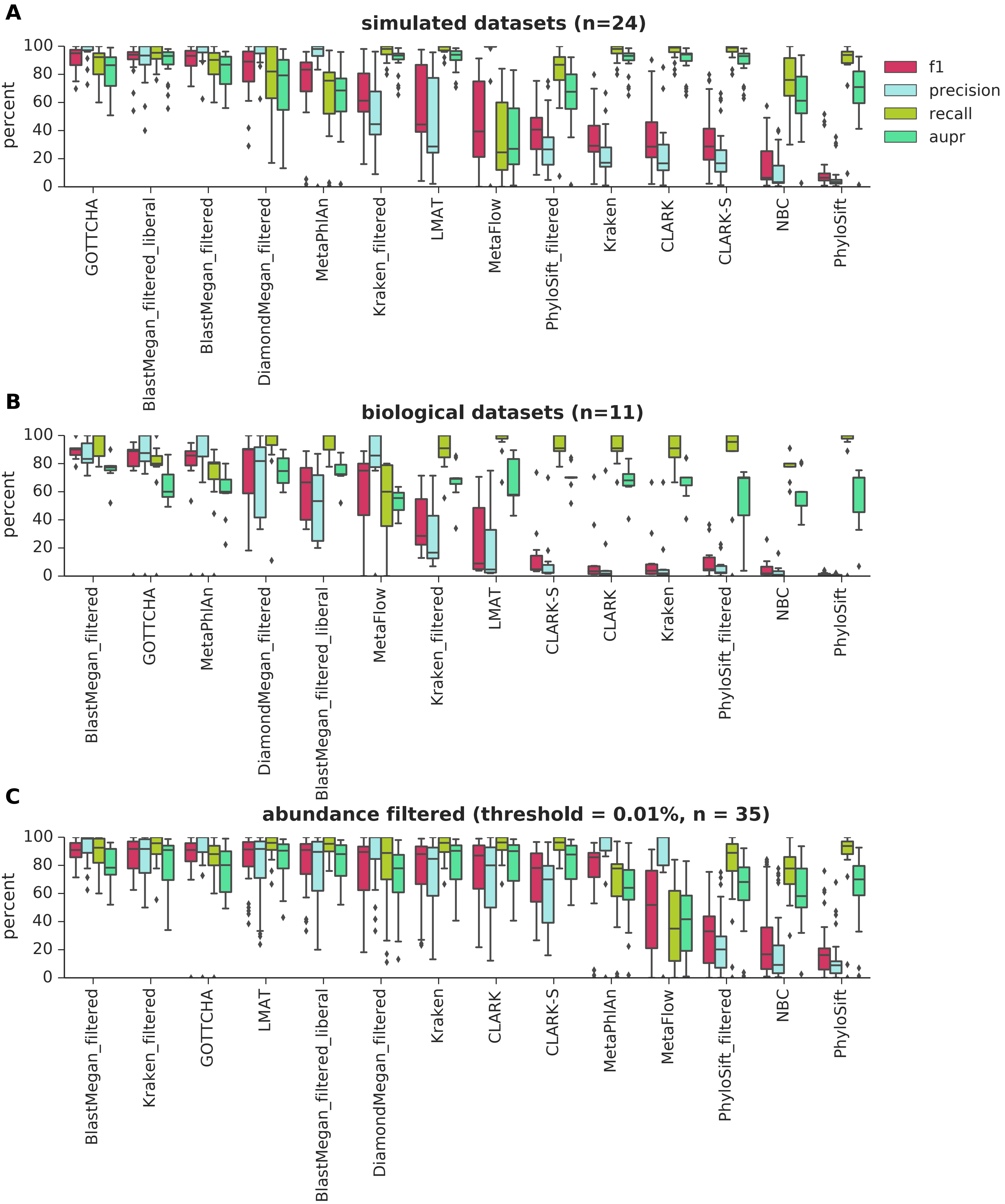

The F1 score, precision, recall, and area under the precision-recall curve for taxonomic classifications at the species level for a) 24 simulated datasets (including three samples of sequenced isolates with reads combined post-sequencing), b) 11 biological datasets, and c) all datasets with an abundance threshold of 0.01%, where tools are sorted by mean F1 score.

Supplementary Figure 2

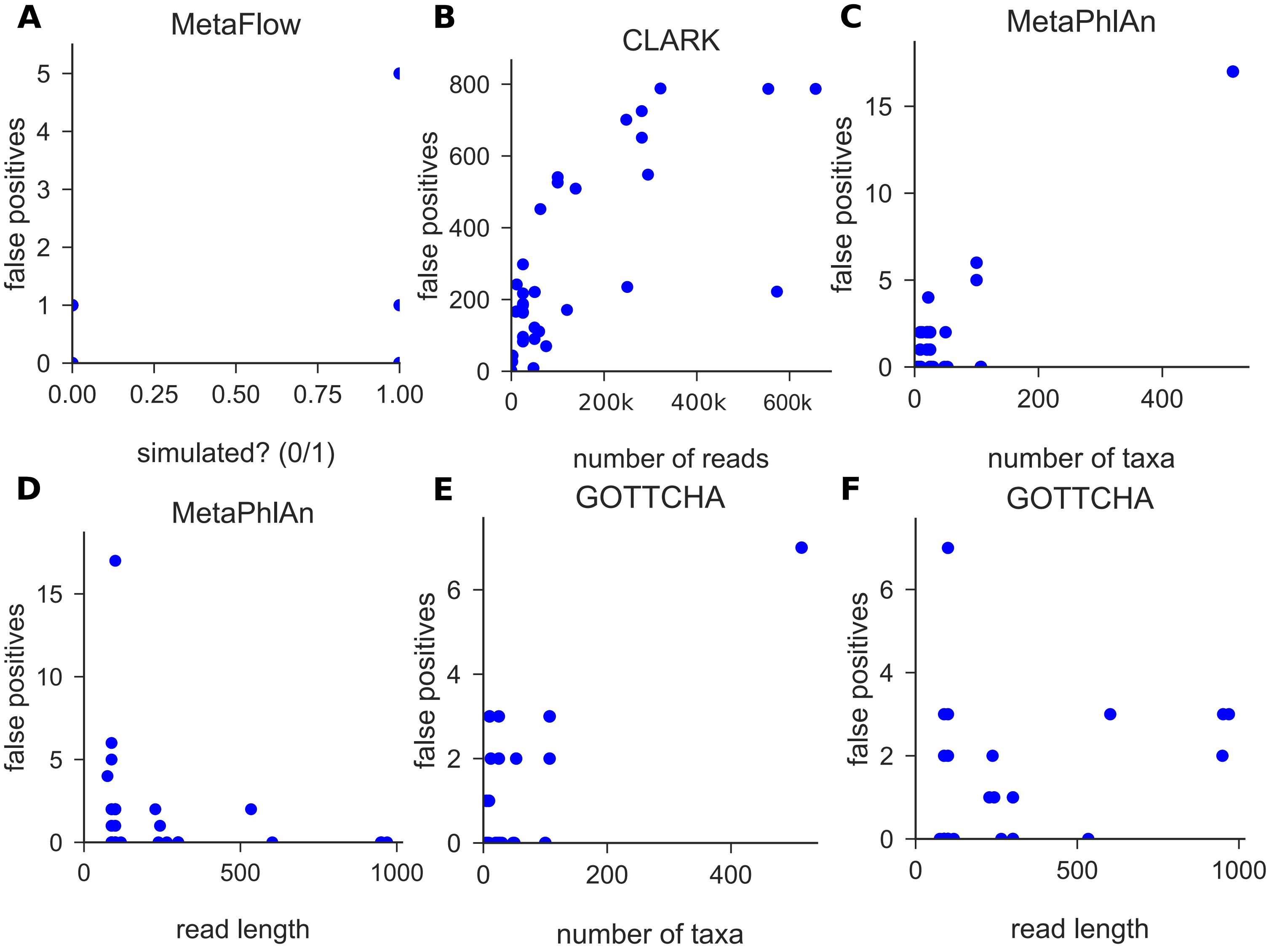

a) Higher false positive rates for MetaFlow decrease for only a subset of simulated datasets. b) False positive rates for CLARK and other k-mer-based classifiers increase as the number of reads in a sample increases. c-d) MetaPhlAn shows significant but noisy relationships between the number of taxa in a sample and read length and the number of false positives it calls. e-f) Similar relationships for GOTTCHA are likely spurious and due to outliers. Additional investigation with more data could help resolve these potential relationships.

Supplementary Figure 3

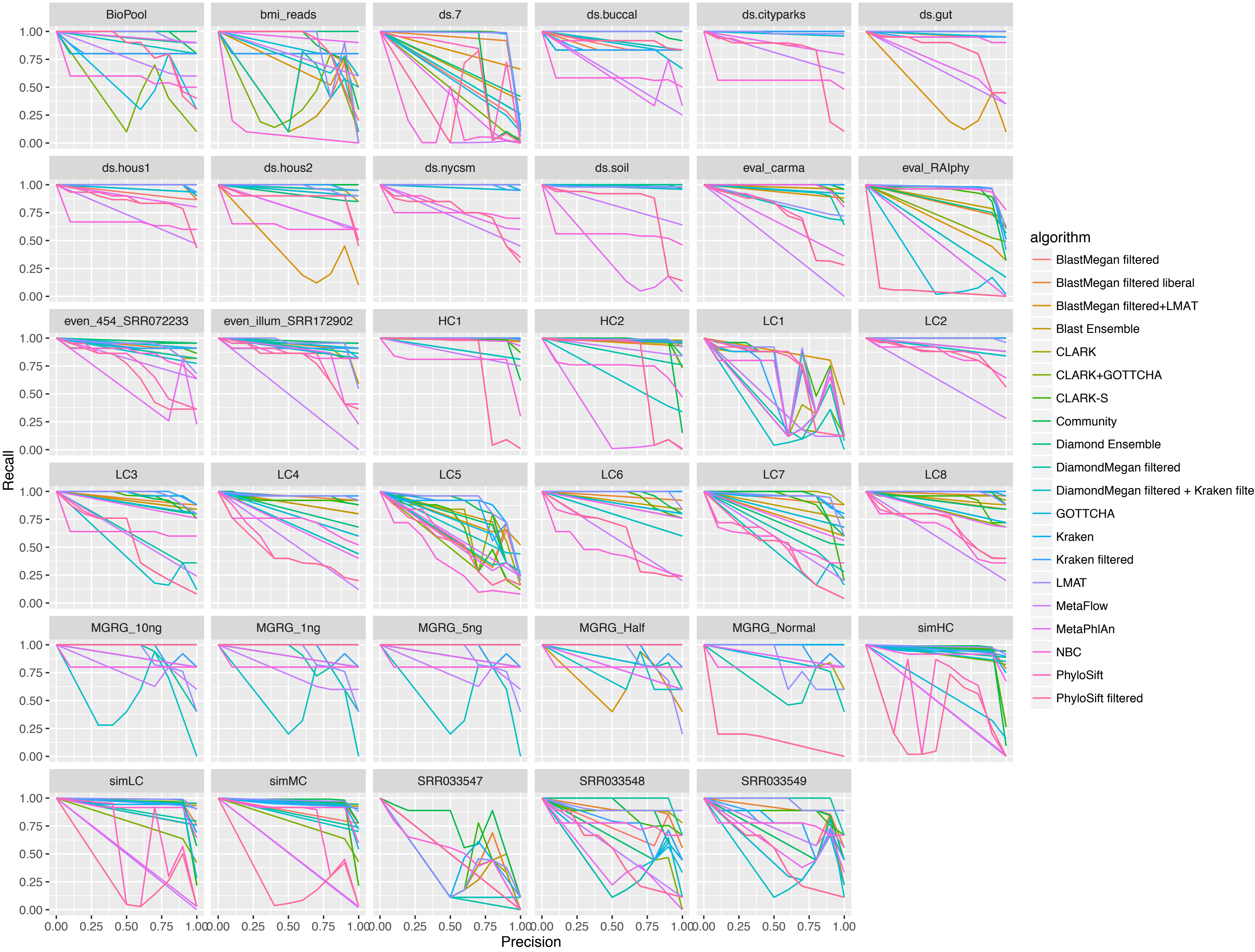

Precision-recall curves for tools on individual samples.

Supplementary Figure 4

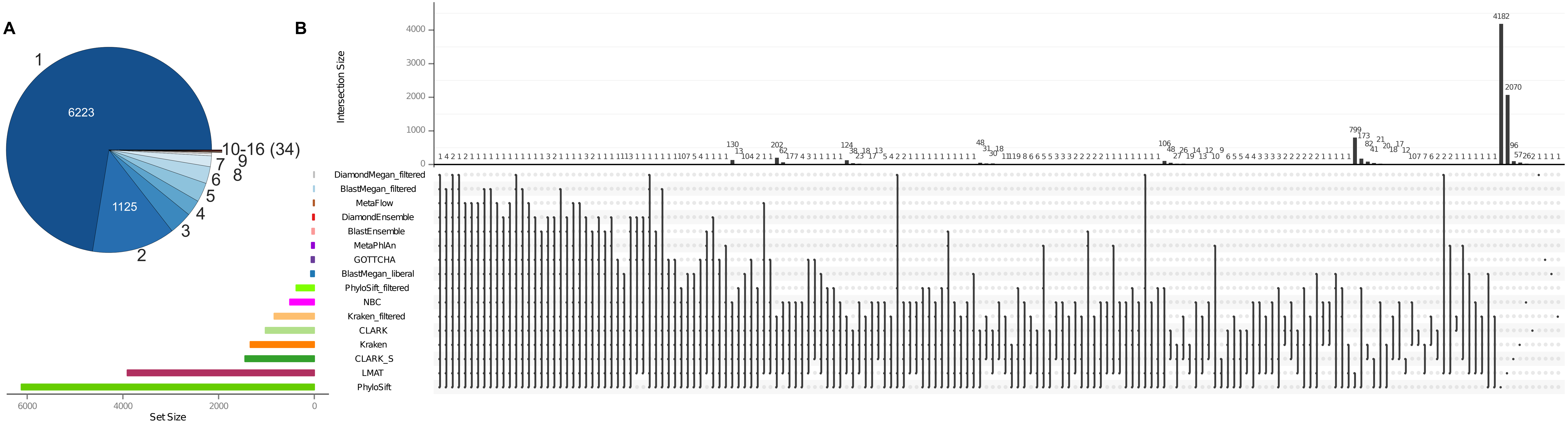

a) The number of species found by between 1 and 16 methods. b) The overlap between species called by different tools and majority vote ensembles for a deeply sequenced (100M reads) sample from the NYC subway system.

Supplementary Figure 5

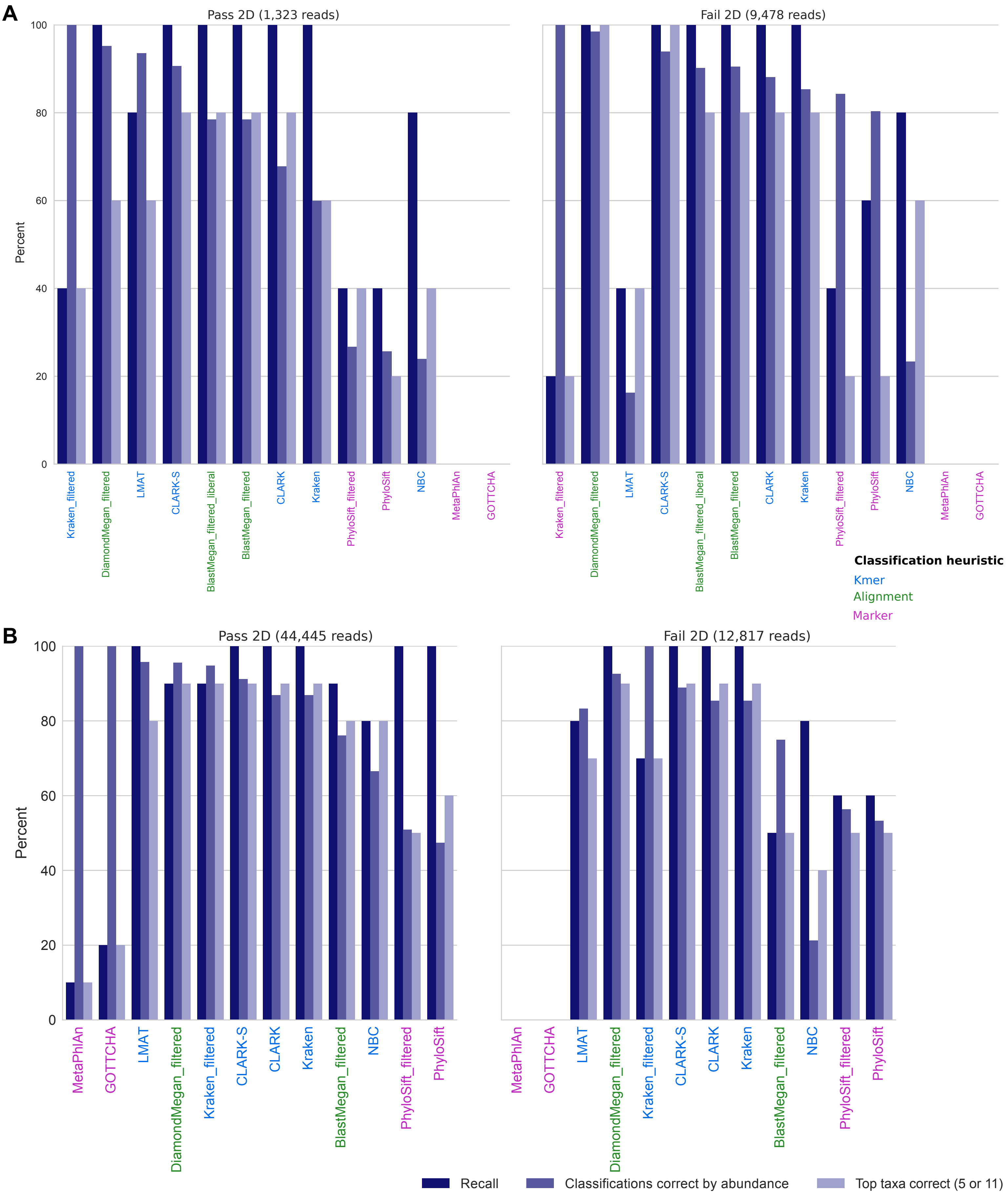

(a) Results from older nanopore data from 2015 for a 5-species mixture. (b) Results from an updated version of the technology with higher throughput and accuracy for an 11-species mixture. Kmer- and alignment-based classifiers attain high accuracy on nanopore data, even with noisy and lower quality ("Fail", average per base quality score < 9) reads, while marker-based strategies are less effective, although this could in part be an issue of coverage. Higher coverage in (b) allows MetaPhlAn and GOTTCHA to correctly identify one or two species. Tools are sorted by the percent of predictions correct by abundance on the 2D pass samples.

Supplementary Figure 6

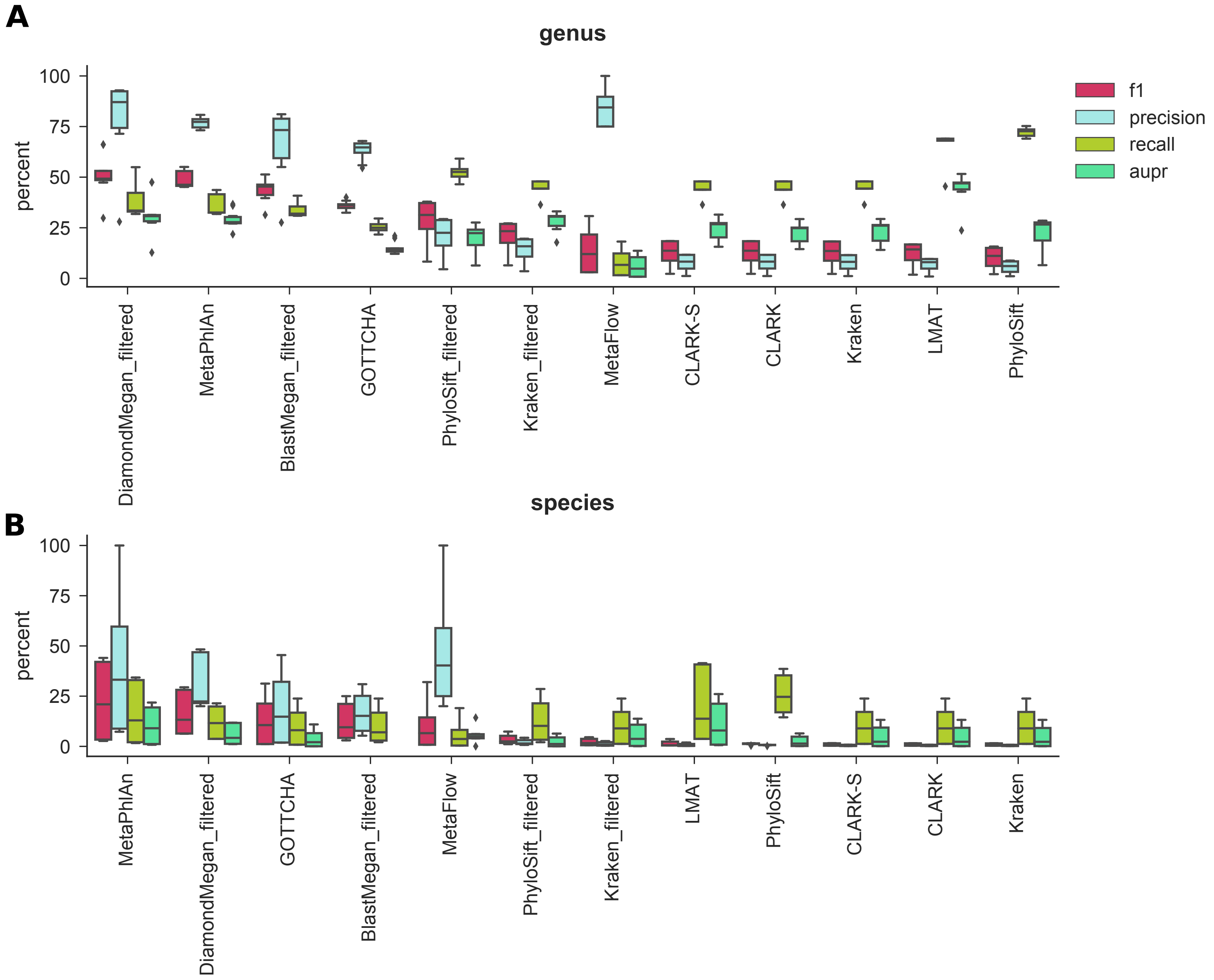

Results at the genus and species levels using simulated datasets from the CAMI study [27] (five “high” complexity, 596 genomes and 478 circular elements; four “medium” complexity, 132 genomes and 100 circular elements; and one “low” complexity, 40 genomes and 20 circular elements). These datasets proved more difficult to identify, but included the added challenges of multiple strains per species, circular elements, and approximately 40% “unidentified species” by abundance.
