# Supplementary material for: Comprehensive benchmarking and ensemble approaches for metagenomic classifiers
Source: Genome Biol. 2017 Sep 21;18:182. doi: 10.1186/s13059-017-1299-7 (PMC5609029; doi:10.1186/s13059-017-1299-7)
Supplement: Supplementary file 9 — Read-level analysis of 21 datasets for seven classifiers and two meta-classifiers that aim to maximize precision and recall, respectively. (DOCX 33 kb) [file 13059_2017_1299_MOESM9_ESM.docx]

| Dataset | Metric | CLARK | CLARK-*S* | Kraken | LMAT | BlastMegan | DiamondMegan | NBC | Meta-Classifier (Precision) | Meta-Classifier (Recall) |
| --- | --- | --- | --- | --- | --- | --- | --- | --- | --- | --- |
| HC1 | Precision | 99.73 | 97.79 | 99.93 | 99.70 | **99.98** | 97.94 | 94.83 | 99.41 (6) | 98.27 (1) |
|  | Recall | 85.10 | **90.30** | 74.16 | 74.57 | 77.38 | 23.92 | 62.42 | 88.91 (6) | 89.49 (1) |
| HC2 | Precision | 99.69 | 96.57 | 99.77 | 99.62 | **99.97** | 97.61 | 93.43 | 99.63 (6) | 97.02 (1) |
|  | Recall | 83.05 | **88.07** | 69.78 | 72.34 | 76.49 | 24.74 | 59.95 | 86.98 (6) | 87.46 (1) |
| LC1 | Precision | 95.42 | 94.23 | 94.36 | 95.84 | 95.39 | 97.55 | 94.75 | **99.93 (7)** | 98.93 (1) |
|  | Recall | 85.89 | **91.05** | 74.57 | 79.90 | 78.25 | 27.91 | 69.88 | 85.44 (7) | 85.78 (1) |
| LC2 | Precision | 99.90 | 99.76 | 99.97 | 99.83 | **99.99** | 98.74 | 99.58 | 99.96 (5) | 99.77 (1) |
|  | Recall | 92.70 | **98.16** | 81.57 | 90.48 | 86.50 | 27.03 | 69.81 | 96.99 (5) | 97.13 (1) |
| LC3 | Precision | 99.81 | 99.48 | **99.96** | 99.52 | 99.93 | 97.62 | 99.53 | 99.94 (7) | 99.45 (1) |
|  | Recall | 92.02 | **96.81** | 80.62 | 62.82 | 85.66 | 23.73 | 74.72 | 95.53 (7) | 95.67 (1) |
| LC4 | Precision | 99.89 | 99.74 | 99.98 | 99.87 | **99.99** | 99.58 | 99.29 | 99.62 (5) | 99.73 (1) |
|  | Recall | 92.56 | **98.28** | 81.44 | 88.17 | 87.08 | 35.99 | 78.86 | 96.68 (5) | 96.83 (1) |
| LC5 | Precision | 96.72 | 93.73 | 87.15 | 95.54 | 90.41 | **98.31** | 91.96 | 98.18 (7) | 94.48 (1) |
|  | Recall | 66.11 | 70.22 | 66.47 | 71.58 | 70.70 | 26.85 | 47.99 | 77.06 (7) | **77.47 (1)** |
| LC6 | Precision | 99.87 | 99.04 | 99.97 | 99.76 | **99.98** | 98.78 | 96.68 | 99.94 (6) | 99.09 (1) |
|  | Recall | 88.98 | **94.67** | 78.38 | 84.33 | 82.57 | 29.61 | 63.00 | 93.36 (6) | 93.55 (1) |
| LC7 | Precision | 99.93 | 98.07 | 99.93 | 99.78 | **99.98** | 98.79 | 99.36 | 99.96 (5) | 98.22 (1) |
|  | Recall | 90.57 | **96.15** | 80.09 | 92.16 | 85.34 | 38.81 | 70.20 | 95.04 (5) | 95.13 (1) |
| LC8 | Precision | 99.73 | 98.77 | 99.94 | 99.77 | **99.98** | 98.79 | 97.52 | 99.84 (6) | 99.12 (1) |
|  | Recall | 84.19 | 89.40 | 78.17 | 83.67 | 83.12 | 25.23 | 68.52 | 93.46 (6) | **93.66 (1)** |
| simHC | Precision | 97.68 | 97.78 | **99.69** | 99.38 | 97.29 | 98.72 | 98.54 | 99.64 (7) | 98.61 (1) |
|  | Recall | 92.54 | 90.81 | 87.91 | 74.83 | 50.16 | 88.99 | 89.96 | 92.27 (7) | **92.58 (1)** |
| simMC | Precision | 98.77 | 98.85 | **99.81** | 99.61 | 98.30 | 99.30 | 99.46 | **99.81 (7)** | 99.23 (1) |
|  | Recall | **95.89** | 95.06 | 93.00 | 86.02 | 56.50 | 94.10 | 93.02 | 95.69 (7) | 95.77 (1) |
| simLC | Precision | 98.61 | 98.62 | **99.79** | 99.61 | 98.24 | 99.20 | 99.43 | 99.74 (7) | 99.10 (1) |
|  | Recall | **95.36** | 94.45 | 92.42 | 84.63 | 56.18 | 93.34 | 93.06 | 95.21 (7) | 95.32 (1) |
| Buc12 | Precision | 95.26 | 93.03 | 98.04 | **98.82** | 95.86 | 98.06 | 98.27 | 98.68 (7) | 95.62 (1) |
|  | Recall | 72.82 | 75.50 | 67.48 | **80.31** | 69.80 | 24.31 | 57.17 | 78.54 (7) | 78.90 (1) |
| CParMed48 | Precision | 99.51 | 99.66 | **99.91** | 99.60 | 99.80 | 99.16 | 98.54 | 99.84 (5) | 99.62 (1) |
|  | Recall | 93.91 | **95.11** | 91.73 | 84.79 | 91.59 | 42.98 | 49.62 | 94.58 (5) | 94.67 (1) |
| Gut20 | Precision | 98.92 | 98.74 | 99.66 | 99.72 | 99.35 | 98.96 | 99.12 | **99.74 (7)** | 99.03 (1) |
|  | Recall | 84.60 | 85.97 | 81.30 | 75.17 | 76.04 | 33.92 | 77.49 | 90.11 (7) | **90.37 (1)** |
| Hous31 | Precision | 97.36 | 97.16 | 98.72 | **99.99** | 97.55 | 98.50 | 97.88 | 98.56 (7) | 98.00 (1) |
|  | Recall | 87.45 | 88.14 | 85.63 | 84.46 | 84.66 | 27.88 | 56.05 | 89.73 (7) | **89.89 (1)** |
| Hous21 | Precision | 99.19 | 99.33 | **99.84** | 96.55 | 99.40 | 98.97 | 99.32 | 99.61 (6) | 99.03 (1) |
|  | Recall | 86.88 | **89.07** | 84.38 | 74.36 | 85.36 | 36.64 | 49.99 | 88.78 (6) | 88.98 (1) |
| NYCSM20 | Precision | 99.23 | 99.07 | 99.71 | **99.92** | 99.60 | 97.68 | 98.82 | 99.78 (6) | 99.35 (1) |
|  | Recall | 85.33 | 85.35 | 80.11 | 68.28 | 69.19 | 24.04 | 59.53 | 90.02 (6) | **90.30 (1)** |
| Soi50 | Precision | 99.51 | 99.47 | 99.89 | 99.65 | 99.72 | 99.05 | 98.53 | **99.83 (7)** | 99.78 (1) |
|  | Recall | 92.86 | 93.61 | 89.28 | 85.62 | 90.58 | 44.26 | 48.74 | 93.71 (7) | **93.76 (1)** |
| simBA525 | Precision | 98.69 | 98.47 | 99.32 | **99.63** | 98.92 | 98.23 | 94.34 | 99.60 (7) | 99.00 (1) |
|  | Recall | 88.63 | 89.10 | 85.30 | 65.62 | 78.18 | 41.63 | 40.30 | 90.93 (1) | **91.32 (1)** |

**Table S7.** Read-level analysis of twenty-one datasets for seven classifiers: CLARK, CLARK-*S* (filtered), Kraken (filtered), LMAT (filtered), BLAST-MEGAN (filtered), Diamond-MEGAN (filtered), and NBC (filtered), as well as the ensemble classifiers (or meta-classifiers) that aim to maximize either precision or recall. Columns 3 to 9 provide the precision and recall for each dataset for all seven classifiers, while column 10 and 11 record the precision and recall of the meta-classifier with the quorum value (in parentheses) that maximizes precision or recall, respectively. Because shorter reads can ambiguously map to multiple species, optimal recall is 100 only for the unambiguously mapping datasets in which all reads should be mapped with high certainty to species.
